# Feasibility of return to sports assessment 6 months after patellar instability surgery

**DOI:** 10.1186/s12891-023-06767-2

**Published:** 2023-08-18

**Authors:** Trine Hysing-Dahl, L. H Magnussen, A. G. H. Faleide, E. Inderhaug

**Affiliations:** 1 Haraldsplass Deaconess Hospital, V/Avdeling for Rehabiliteringstjenester Postboks 6165, Bergen, 5892 Norway; 2 Haukeland University Hospital, Bergen, Norway; 3 Western Norway University of Applied Science, Haugesund, Norway; 4 University of Bergen, Bergen, Norway

**Keywords:** BPII 2.0, Functional tests, Lateral patellar dislocation, NPI, Patellar instability, Return to sports

## Abstract

**Background:**

The evidence regarding the usefulness of assessment tools to support decisions of return-to-sport after surgery for patellar instability is scarce. The purpose of this study was therefore to explore the feasibility of functional tests assessing readiness for return-to-sport six months after patellar stabilizing surgery. However, there is little evidence on what a functional assessment should include to support these decisions following surgery for patellar instability. Therefore the purpose of this study was to explore the feasibility of functional tests assessing readiness for return-to-sport six months after patellar stabilizing surgery.

**Methods:**

In this cross-sectional study a prospective cohort of 78 patients were subjected to a range of return-to-sport readiness tests at six months after surgery for patellar instability with an “a la carte” approach. Lower Quarter Y-Balance Test (YBT-LQ), single-legged hop tests and isokinetic strength tests were performed. In addition, self-reported function was measured with the Banff Patellofemoral Instability Instrument 2.0 (BPII) and Norwich Patellar Instability score (NPI). Return-to-sport clearance criteria were defined as: ≤4 cm YBT-LQ anterior reach difference between legs, leg-symmetry-index (LSI) ≥ 95% in the YBT-LQ composite score, mean sum score LSI ≥ 85% of all single-leg hop tests and LSI ≥ 90% in isokinetic quadriceps strength.

**Results:**

Sixty-four patients (82%) were able to complete all functional tests, while only eleven (14%) patients were deemed ready for return-to-sport, passing all return-to-sport clearance criteria. Patients with bilateral problems demonstrated worse performance in the contralateral leg, which resulted in higher LSI scores compared to individuals with unilateral instability. A supplementary finding was that the extent of surgery (MPFL-R only versus combined surgery) did not predict and mainly did not affect self-reported function or functional performance at the follow-up.

**Conclusion:**

The functional assessment used in the current study seems feasible to conduct at six months after patellar stabilizing surgery. However, current suggested clearance standards and the use of leg-symmetry-index seems inappropriate for patients with patellar instability. Therefore, further exploration of appropriate tests and return-to-sport clearance criteria is justified.

**Trial registration:**

clinicaltrial.gov, NCT05119088. Registered 12.11.2021 - Retrospectively registered, https://clinicaltrials.gov/ct2/show/NCT05119088.

## Background

There is a broad agreement that patients with recurrent patellar dislocations who do not achieve satisfactory function with rehabilitation should be offered surgery [1–5]. A common approach is to address each patient’s deviant knee anatomy. This so-called “a la carte” method includes procedures such as tibial tubercle realignment, trochleoplasty and/or derotational osteotomies in addition to medial patellofemoral ligament reconstruction (MPFL-R) [6].

The aim of surgery is to stabilize the patella so that patients can regain knee function and participate in the activities/sports they desire. The postoperative rehabilitation is often long and demanding and six months after surgery patients may start to consider returning to sport (RTS) or other knee-challenging activities [7]. It would therefore be helpful to evaluate physical function and RTS readiness at this time point to advise patients on whether they are ready to challenge their knee in sport again or whether they should “hold back” and continue rehabilitation.

Some studies have reported the use of functional evaluations comprising various tests [8–13] and expert groups have proposed RTS clearance criteria for patients with patellar instability (PI) including criteria such as no pain, no effusion, no patellofemoral instability, a full range of motion, nearly symmetrical strength, and excellent dynamic stability [3, 14]. These are often inspired by methods applied on patients with anterior cruciate ligament (ACL) injury, calculating Leg Symmetry Indexes (LSIs) from hop and strength tests to compare function of the involved leg to the contralateral leg. However, at this point, there is little evidence on what a functional assessment should include to support the RTS decision following surgery – what tests and criteria will provide the information we seek to advise the patients [3, 15–17]. Moreover, there is little knowledge about the validity of using such tests for this patient group [16]. Suggested tests and “clearance standards” therefore need further clinical evaluation to ensure the appropriateness for patients after patellar stabilizing surgery, especially since some of the RTS clearance criteria include the use of LSIs in a group of patients where many have bilateral problems.

The aim of the current study was therefore to explore the feasibility of functional tests assessing readiness for RTS six months after surgery for recurrent patellar dislocation, by examining (1) how many patients who were able to complete the tests, (2) achievability of suggested clearance standards for RTS and (3) appropriateness of LSI measures for patients with PI.

## Materials and methods

From January 2021 to December 2022, patients undergoing surgical treatment for recurrent (two or more) patellar dislocation were prospectively recruited from three Norwegian Orthopaedic Centres; Haukeland University Hospital, Haraldsplass Deaconess Hospital and Laerdal Hospital. Inclusion criteria were 13 to 45 years at surgery and fluency in Norwegian. Patients with concomitant knee injuries were excluded. Written informed consent was obtained from all patients prior to data collection. For patients under 18 years, legal guardians signed the consent. The study protocol was retrospectively registered and is available at ClinicalTrials.gov (NCT05119088). The study was approved by the Norwegian Centre for Research Data, Data Protection Official for Research, project number 731,409 and the Regional Committee for Medical and Health Research Ethics (ID: 2020/185,067).

### Surgical procedures

Prior to surgery, all patients had been advised to undergo an exercise program targeting neuromuscular deficits. Type of surgery was based on findings from the preoperative counselling and radiologic examinations, including radiographs and MRI scans. All patients underwent a MPFL-R by use of a gracilis autograft from the ipsilateral knee. The tendon was inserted in the medial proximal patella through two connected anterior drill holes. Further, the tendon was tunnelled down to its femoral insertion and secured with a PEEK interference screw (Arthrex, Naples, US).

Tibial tubercle osteotomy with distalisation or medialisation was considered in cases of patella alta or in patients with a lateralisation of the patella, measured by the tibial tuberosity- trochlear groove distance (TTTG). Elevated TTTG from 15 to 20 or Caton-Deschamps Index above 1.3 was typically considered an indication for these procedures either alone or in combination.

Finally, a trochleoplasty was considered in cases of a severely dysplastic patella. Typically, Dejour type B and D dysplasia with a proximal bump and/or a lateral trochlear index of less than 11^o^ were considered for surgery. A semi-open thin-flap technique was performed through a lateral parapatellar incision. One or two bioabsorbable SmartNail implants (ConMed, Utica, US) were then used to create the new groove of the trochlea.

### Postoperative treatment

General advice on early neuromuscular exercises was given upon discharge from the day-care unit, and all patients conducted postoperative rehabilitation with their local physiotherapist. Patients did not wear a brace and were allowed foot-touch weight-bearing from the first postoperative day supported by crutches for six weeks. From four weeks postoperatively, patients were allowed gradual full weight-bearing until weaning off crutches.

### Readiness assessment

The International Classification of Functioning, Disability and Health was used as a framework to ensure the selected outcome measures evaluated relevant aspects of patients knee function [18]. To capture patients’ subjective function, including mental readiness for RTS, Banff Patellofemoral Instability Instrument 2.0 (BPII) and Norwich Patellar Instability score (NPI) were included. The functional tests used for readiness assessment were selected based on two former expert recommendations [3, 14]. All patients were evaluated six months postoperatively. At the day of testing, questionnaires were completed before participants completed a seven-minute warm-up on a stationary bike and underwent the functional tests. All patients were evaluated by the same, independent, examiner not formerly involved in their treatment.

### Patient reported outcome measures (PROMs)

The BPII 2.0 is a self-administered, disease-specific quality of life (QOL) score that consists of 23 questions covering five domains: symptoms/physical complaints, work-related concerns, recreational activity and sports participation [19]. Patients grade their answers on a 100 mm VAS scale. A total score is calculated as the average of the responses on each question, range 0-100, where higher scores indicate better QOL [19]. The Norwegian version of the BPII 2.0 is valid and reliable for patients with PI [20].

The NPI score is a 19-item score of self-experienced PI during activity [21]. Patients respond using a five-point Likert scale with options from “never” to “always” [21]. The score is presented as a mean percentage where a higher score indicates more instability. The NPI has demonstrated good measurement properties in several domains [21–23], and has recently been translated into Norwegian.

### Functional tests

The *Lower Quarter Y-balance Test* (YBT-LQ) evaluates lower extremity strength, knee stability and dynamic balance in anterior, posteromedial and posterolateral direction [24]. For each direction, three practice trials were allowed before three test trials were recorded. Mean reach distances (in centimetres, (cm)) was normalized to leg length, which was measured from the anterior superior iliac spine to the most distal portion of the medial malleolus. The results are presented as normalized reach values in anterior direction, difference in anterior reach distance (cm) between legs, and a composite score determined using the following equation:

*Composite score = [anterior + posteromedial + posterolateral) / (3 x leg length)] x 100*

The YBT-LQ has shown predictive validity for injury risk and is a reliable test for measuring single leg dynamic balance [24, 25].

The *single-legged hop test* evaluates functional performance, dynamic strength and lower extremity muscle power [3, 12]. It comprises four tasks: a single hop for distance (cm); triple hops for distance (cm); triple crossover hops for distance (cm); and 6-m timed hops (in seconds) [26]. One practice trial on each hop test was performed before two test trials were completed. No rest was allowed between tests. The results are presented as a mean of the two test trials in absolute values (cm), and a mean LSI%; (involved leg/contralateral leg) x 100%) of the four tests. A score of 100% meant there was complete symmetry in the performance of the legs. Values < 100% indicated a deficit in the involved leg [24, 25]. Hop tests are reliable and valid for patients with other knee injuries such as ACL rupture [27].

*Concentric muscle strength* was evaluated at 60^o^/Sect. (5 repetitions) angular velocity using an isokinetic device (Biodex system 4 dynamometers, Biodex Medical Systems Inc.). Performance was presented as absolute values (in Newton meters (Nm)), and peak torque (PT) LSI% [28]. Isokinetic strength tests have been found to be a reliable measure of muscle strength after other knee injuries and are considered the ‘gold standard’ for measuring muscle strength [28, 29].

“Results from each functional test was normalized to z-scores (z = x – population mean/population standard deviation) and then added, creating a new “performance at six months” composite variable. The approach of adding z-scores to make a single composite score has not been used extensively, but may have its benefit to represent a broader construct of physical performance in PI-patients [30].

### RTS clearance criteria

RTS clearance criteria for the functional tests were defined as previously suggested for patients with PI [3, 14]: LSI ≥ 95% composite score for the YBT-LQ, ≤ 4 cm YBT-LQ anterior reach difference between legs, LSI ≥ 85% for all single-leg hop tasks and LSI ≥ 90% in quadriceps strength [3, 12–14, 31]. The BPII and NPI were a supplementary part of the RTS assessment and not included in the RTS clearance criteria as no evidence exists regarding clearance values for these two PROMs.

### Statistical analyses

All statistical analyses were performed using the IBM SPSS Statistics for Windows, version 26.0 (IBM Corp). As this is a feasibility study the focus was on the feasibility of the current assessment and no formal power analysis were performed. The a priori significance level was set to ≤ 0.05. Descriptive analyses were expressed as mean ± SD for continuous variables and frequencies and percentages for categorical variables. Independent samples t-tests were conducted to investigate differences in [1] PT between the legs, [2] reach distance and composite score between the legs on YBT-LQ and hop tests and [3] differences in performance based on bilateral problems and extent of surgery. To examine which factors that predict performance, backward multiple regression was performed. With performance six months postoperative as the dependent variable, age, gender, extent of surgery, duration of symptoms and bilateral/unilateral problems was entered as independent variables, and only variables with a p-value ≤ 0.10 were included in the final model. Multicollinearity was assessed by inspecting the tolerance values in linear regression analysis, and values < 0.1 were interpreted to indicate correlations that are too high between variables [32].

## Results

### Patient demographics

**Fig. 1:**
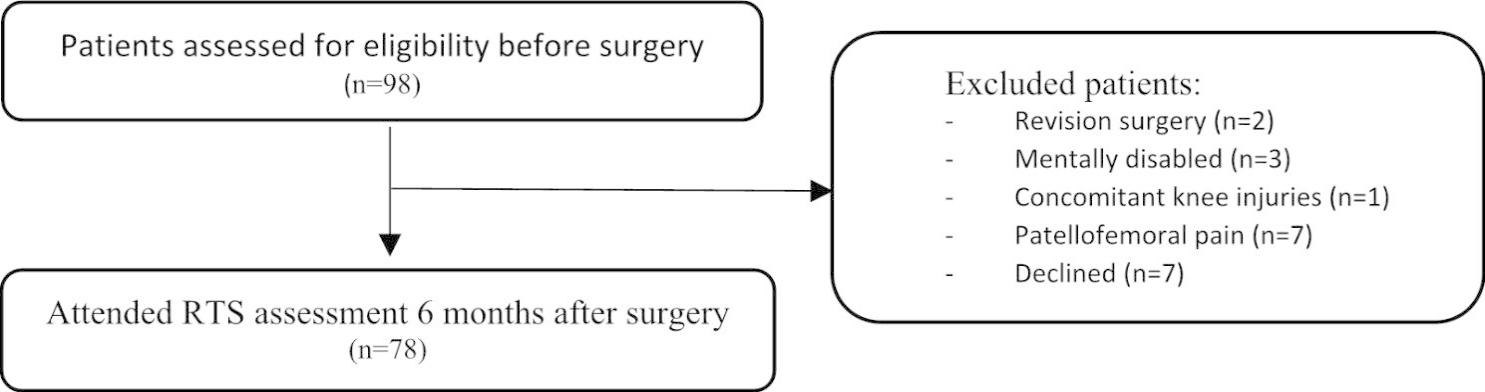
Flowchart of patient’s participation

Of 98 patients screened for eligibility, 78 patients (71% female, mean age 22.3 ± 6.9 (range 13–45 years), BMI 25.3 ± 5.2) were enrolled in this study after exclusions (Fig. 1). Mean time since first dislocation was 7.0 years (± 5.9), and 60% reported bilateral problems. Functional testing was performed on average 6.1 months (± 0.8) after surgery. 19% (n = 15) of patients underwent an isolated MPFL-R while 81% (n = 63) underwent combined surgery (including either TTO and/or trochleoplasty). Pre-surgery level of activity/sports were competitive in 38% of the patients and recreational in 62%. Mean BPII score was 65.1 (± 19.9), and mean NPI score was 9.9 (± 11.3) at that follow-up.

### Ability to complete the tests

Sixty-four patients (82%) were able to complete *all* functional tests at the six-months assessment. Looking at the tests separately, all - but one - completed the YBT-LQ test, 64 patients (82%) completed the hop tests, and all patients completed the isokinetic strength testing. Performance was generally impaired on the involved leg compared to the contralateral leg (see Table 1).

**Table 1:** Performance and pass rates on functional tests six months after surgery (n = 78)^a^

Test	Involved Leg	Contralateral Leg	LSI, %	*P* Value	*Passed RTS criteria, % (n)*
YBT-LQ, Composite score	74.3 ± 9.3	77.3 ± 9.0	96.1	**0.047**	64.1 (50)
YBT-LQ, Normalized reach (%) anterior	65.1 ± 7.3	68.7 ± 6.6		**0.001**	
Single hop for distance, cm	72.3 ± 34.2	85.4 ± 30.2	82.0	**0.016**	50.0 (39)
Triple hop for distance, cm	273.2 ± 100.2	305.1 ± 88.2	88.4	0.051	55.1 (43)
Crossover hop for distance, cm	240.4 ± 94.4	255.5 ± 98.4	95.8	0.374	62.8 (49)
6-m timed hop, s	3.3 ± 1.3	3.1 ± 1.1	95.3	0.367	64.1 (50)
LSI ≥ 85% all 4 hop test			91.0		42.3 (33)
PT extension 60°/s, Nm^b^	92.2 ± 41.4	130.9 ± 46.5	72.0	**0.001**	19.2 (15)
PT flexion 60°/s, Nm^b^	64.6 ± 23.2	69.1 ± 23.1	94.3	0.241	63.0 (34)

^a^Data are reported as mean ± SD unless otherwise specified. Bolded P value indicates a statistically significant difference between the legs (P ≤ .05). YBT-LQ, Lower Quarter Y-Balance test, LSI, Leg Symmetry Index, PT, Peak Torque

^i^Information missing in 3 patients n = 75

### Achievement of suggested clearance standards for RTS

In total eleven patients (14%) passed *all* the RTS clearance criteria and were therefore deemed ready for sport resumption. In the YBT-LQ test, 64% passed the return criteria (composite score LSI ≥ 95% and anterior reach asymmetry ≤ 4 cm). For the four hop tests, a mean sum score of 91% LSI was seen across all patients – but only 33 patients reached the RTS clearance criteria (LSI ≥ 85%) for this test. On the isokinetic strength test, the mean LSI was 72% across all patients - and only 19% of patients achieved the RTS clearance criteria (LSI ≥ 90%) (Table 1). The eleven patients who passed all RTS clearance criteria more often had bilateral problems and were of younger age (mean 17.4 vs. mean 22.3 years) when compared to the other patients. Their pre-surgery level of activity/sport was equal to the rest of the cohort.

### Measures of leg symmetry index

Those with *bilateral* problems had higher absolute LSI scores on all functional tests compared to individuals with unilateral problems (Table 2) – but only the hop test LSI sum score and anterior reach difference between legs reached statistical significance (*P* ≤ .05). Comparing the *contralateral leg* of those with *bilateral instability* to the *contralateral leg* of those with *unilateral instability*, patients with bilateral instability demonstrated worse performance in knee extension strength and crossover hop distance on what would be defined as the “healthy leg” when calculating LSI’s (Table 2). This illustrates how patients with bilateral patellar instability have reduced leg strength and hop ability in *both legs* and therefore is it problematic to use the contralateral leg as a «gold standard» in these patients. No difference in *contralateral leg* performance was found for knee flexion strength, the YBT-LQ test or the other hop tests (*P* > .05).

**Table 2:** Functional performance *of contralateral leg* in patients with uni- compared to bilateral patellar instability (n = 78)^a^

Test	All patients	Bilateral problems	Unilateral problems	*P* Value
YBT-LQ anterior reach difference, cm	3.2 ± 3.8	2.4 ± 3.3	4.5 ± 4.1	**0.017**
YBT-LQ, Composite score, LSI, %	96.4 ± 5.6	96.9 ± 5.9	95.6 ± 5.1	0.368
Mean sum score hop test LSI, %	90.8 ± 15.6	96.7 ± 12.8	83.8 ± 15.9	**0.001**
PT extension 60°/s, Nm, LSI, %	72.0 ± 25.1	76.6 ± 28.2	65.5 ± 18.6	0.060
YBT-LQ, Composite score	77.3 ± 9.0	77.4 ± 9.2	76.9 ± 9.0	0.796
Single hop for distance, *contralateral leg*, cm	85.4 ± 30.2	84.6 ± 29.6	86.4 ± 31.4	0.812
Triple hop for distance *contralateral leg*, cm	305.1 ± 88.2	294.8 ± 81.4	319.5 ± 96.6	0.267
Crossover hop for distance *contralateral leg*, cm	255.5 ± 98.4	234.3 ± 78.4	289.5 ± 117.9	**0.045**
6-m timed hop *contralateral leg*, s	3.1 ± 1.1	3.2 ± 1.0	3.0 ± 1.2	0.485
PT extension 60°/s, Nm, *contralateral leg*^a^	130.9 ± 46.5	119.1 ± 43.1	147.7 ± 46.6	**0.008**
PT flexion 60°/s, Nm, *contralateral leg*^a^	69.1 ± 23.1	67.1 ± 22.4	72.0 ± 24.1	0.372
Performance composite (z-score)	0.003 ± 0.68	0,05 ± 0.10	-0.06 ± 0.12	0.499

^a^Data are reported as mean ± SD unless otherwise specified. Bolded P value indicates a statistically significant difference between groups (P < .05). LSI, leg symmetry index, PT, Peak Torque

^a^Information missing in 3 patients n = 75

### The extent of surgery

The extent of surgery (MPFL-R only versus combined surgery) affected only normalized anterior reach distance in involved (68.5 ± 5.5 vs. 64.2 ± 7.5; *P* = .04) and contralateral leg (71.5 ± 4.0 vs. 68.0 ± 7.0; *P* = .01), but the correlation was minor (-0.234, *P =* .04 and − 0.208, *P =* .06). No other statistically significant difference in functional tests or the PROM scores were seen between patients with MPFL-R only versus combined surgery at the six months assessment (All *P* > .05).

### Predictors of performance

In the backward multiple regression, age and gender remained independent significant predictors of performance six months after surgery, with a shared explained variance of 21% (Table 3). Tolerance values were both 0.98, indicating no problems with multicollinearity.

**Table 3:** Prediction of performance at six months postoperatively. Final multiple regression models after backwards elimination with gender, age, type of surgery, duration of symptoms and bilateral/unilateral problems as covariates (n = 78)

Dependent Variable	Independent variables	B (CI)	Beta	p-value^*^	R^2^
Composite performance (z score)	Gender	− 0.314 (-0.627,-0.002)	− 0.211	0.049	
	Age at surgery	− 0.037 (-0.057, − 0.016)	− 0.375	0.001	0.208

*Independent variables predicting performance six months postoperatively with p ≤ .10 were retained in the final model. CI, confidence interval

## Discussion

The most important finding from the current study – evaluating functional tests six months after surgery for recurrent patellar instability – was a high degree of completion across the different tests. Although completion rates were high, only eleven out of 78 patients passed all the RTS clearance criteria suggested in current literature. Because patients with bilateral problems demonstrated impaired performance in the contralateral leg, they also displayed higher LSI scores than individuals with unilateral instability. A supplementary finding was that the extent of surgery (MPFL-R only versus combined surgery) mainly did not affect self-reported function or functional performance and only gender and age at surgery predicted performance six months postoperatively.

Most patients were able to perform all tests in the current study when evaluated six months after surgery, indicating that the tests are appropriate for this patient group. However, only 14% met all the RTS clearance criteria at this time point. In comparison, Matassi et al. [10] found a 40% readiness clearance rate at 8 months after MPFL-R. That study, however, applied a slightly different test battery including balance, strength, speed and agility tests – at a later time in the rehabilitation process (range 8–35 months). On the hop-tests, 42% passed all four tests in the current cohort. In comparison, Saper et al. [12] described lower hop-test pass rates (32%) at 7 months after stabilizing surgery in a study including adolescents with unilateral instability only. As illustrated, discrepancies in findings might be due to several factors, such as timing of the RTS evaluation, the type of tests applied and surgical approach. Further comparisons across available published RTS data are therefore difficult.

The current finding of 60% bilateral leg involvement in patients with PI is in line with other reports [33]. As calculating an LSI involves using the contralateral – assumingly “normal” – leg as a reference, its use can be erroneous for patients with PI. Overall, the quadriceps strength in the contralateral leg (not the one that had undergone surgery) was significantly reduced in those with bilateral, compared to those with unilateral instability. When applying LSI, this discrepancy will give the impression of a symmetrical performance and thereby a satisfactory outcome - when in fact - both legs might have inadequate muscle strength. The reporting LSI’s adapted from assessment of patients with ACL injury therefore seems inappropriate for patients with PI. Evaluation of absolute values and comparison to normative references populations can - to a certain degree - overcome this issue. Serial measurements of the same leg over time can also contribute to a more appropriate functional evaluation of patients with PI.

Performance on functional tests revealed that the patients in the current study have pronounced functional limitations six months after surgery. This is in line with other studies reporting persistent reduced knee function after surgery for recurrent patellar dislocation [8, 9]. It is indicated that an anterior reach asymmetry of ≥ 4 cm on the YBT-LQ test may predict an increased risk of future lower extremity injury [34]. The patients who had asymmetry of ≥ 4 cm, approximately one out of three in the current cohort, may therefore return to sport with an increased risk of further injuries. The isokinetic strength deficits at six months seen in the current and previous studies [12, 13, 35], also implies that patients may not be able to generate the forces needed to stabilize the knee and maximize functional ability, and therefore need more strength training before returning to knee-challenging activities. Interestingly, a recent systematic review reported that more than 90% of patients with PI resumed athletic activity at a mean of 6.7 months after surgery [36]. Based on our experience from the present study, returning to sport at six months seems premature and it may be more appropriate to have the patients exercise more before conducting RTS assessments *nine* months after patellar stabilizing surgery - a time that is well established after ACL reconstruction [37]. This recognition should inspire further investigations of the timing of RTS assessment and maybe more structured exercise programs for the late phase rehabilitation in patients with PI.

The current RTS clearance criteria are similar to widely used criteria applied on patients with an ACL tear [38–40]. Although similarities exist in injury mechanism and neuromuscular deficits between those patient groups, comparison should be done with caution. The amount of patients with bilateral problems is lower, participation at competitive level of sports is higher [40–43] and physical performance is generally better in patients with ACL tears [38, 40, 43]. Faleide et al. [40] reported that 69% of patients with ACL reconstructions competed in elite to lower competitive levels while 31% participated at a recreational level of activity. Other ACL studies report an even higher share of patients performing sports at competitive levels [37, 42]. In contrast, the current study had 38% competing in elite, competitive or lower competitive level while 62% participated in recreational activities only. Furthermore, the current cohort had long-standing symptoms and a mean of 7 years from first dislocation to surgery - indicating that PI should be regarded as a more chronic condition than an ACL tear. This illustrates that the two patient groups are not directly comparable and thereby emphasize how patients with PI need different tests and clearance standards. Further, one may question whether RTS testing is relevant to this heterogeneous population at all. Perhaps is only a selection of patients (aiming to return to pivoting activities) in need of functional RTS testing, while the majority might be better off with a return to *activity* assessment with less demanding clearance standards than those applied for athletes.

When adopting RTS assessment from ACL research to patients who have undergone surgery for PI, it is interesting to note that psychological readiness has not been addressed in any of the former PI-studies [10–12]. The impact of kinesiophobia and mental readiness for resuming sport are increasingly documented after ACL injuries [40]. Lack of mental readiness for challenging the knee, may also presumably play an important role after patellar stabilization surgery. The two diagnose-specific PROMs included in the current both addresses some psychological factors, and the results indicated that psychological readiness was affected in the current population. This is in line with findings by Platt et al. [36] and Hurley et al. [44] which indicated that the most common reason for patients choosing to lower their level of sport participation after MPFL-R was fear of new dislocations. However, as no clearance standards for the BPII and the NPI exists, future work is warranted to enhance interpretation of the questionnaires and possibly implement them in RTS evaluations.

This far, research on patients with PI has been conducted on relatively small and homogeneous cohorts, often undergoing uniform surgical procedures [8, 10–12]. Due to the diversity in selected populations and surgical procedures it is difficult to compare across studies. All patients in the current study underwent surgery with an “a la carte” approach making the results relevant to a broad spectrum of patients. Our results may, however, not be relevant for competitive athletes following extensive rehabilitation protocols at specialized clinics. Furthermore, one might argue that including patients who have undergone differing procedures in one study pose some challenges. While a recent systematic review reported that combined surgery did not affect time to RTS [36], Krych et al. [13] reported that patients who had undergone combined procedures had inferior quadriceps strength compared to those who had undergone isolated MPFL-R. Interestingly, the extent of surgery in the present study, did not affect neither the PROM scores nor performance on functional tests, except YBT-LQ normalized anterior reach – indicating that patients with differing surgical procedures can be evaluated in the same cohort. This is further supported by the finding that neither the extent of surgery, bilateral/unilateral instability nor the duration of symptoms predicted performance, while gender and age predicted performance. This might not be surprising as it is well-known that performance vary between men and women, and performance is assumed to decrease with increasing age.

All the current patients underwent postoperative rehabilitation, but no standardized rehabilitation protocol was applied - and rehabilitation was performed in several different locations. It is therefore unclear how a potential heterogeneity in rehabilitation might have affected the current outcomes. Future studies should try to control such variables. Other limitations in this study includes no data on number on dislocation episodes and the skewed distribution between genders where the majority of patients were female. This reflects the population experiencing PI as females more often experience this disorder. However, results should be interpreted with this in mind.

## Conclusion

The functional assessment used in the current cohort was feasible to conduct at six months after patellar stabilizing surgery. However, achievability of current suggested return-to-sport clearance standards was low and the use of leg symmetry index measures seems inappropriate for patients with patellar instability due to the high proportion of patients with bilateral complaints. More knowledge is needed on what tests to use, the timing of their use - and the level of performance that suggests readiness for return to sport. In addition, one should consider if a majority of this highly heterogeneous group of patients might be better off with a *return to activity* rather than *return to sports* assessment. Our findings indicate a need for further refinement of readiness assessment for patients with patellar instability.

## Data Availability

The datasets used and analysed during the current study are available from the corresponding author on reasonable request.

## Abbreviations

ACL: Anterior cruciate ligament
BPII: Banff Patellofemoral Instability Instrument 2.0
CM: Centimetres
LSI: Leg Symmetry Indexes
MPFL-R: Medial patellofemoral ligament reconstruction
NM: Newton meters
NPI: Norwich Patellar Instability score
PI: Patellar instability
PROMs: Patient Reported Outcome Measures
PT: Peak torque
QOL: Quality of life
RTS: Return to sport
TTTG: Tibial tuberosity- trochlear groove
YBT-LQ: Lower Quarter Y-balance Test
